# Off-Pump Coronary Artery Bypass Grafting With Nafamostat in a Patient With Kasabach-Merritt Syndrome: A Case Report

**DOI:** 10.7759/cureus.106489

**Published:** 2026-04-05

**Authors:** Yosuke Ito, Akiko Yoshida, Kei Nagaya, Shin Kurosawa

**Affiliations:** 1 Anesthesiology, Tohoku Medical and Pharmaceutical University, Sendai, JPN

**Keywords:** consumptive coagulopathy, heparin contraindication, kasabach-merritt syndrome, nafamostat mesilate, off-pump cabg

## Abstract

Heparin is used as an anticoagulant in coronary artery bypass grafting (CABG), but in conditions where heparin is contraindicated, an alternative anticoagulant is required. Kasabach-Merritt syndrome is characterized by thrombocytopenia and consumption coagulopathy associated with kaposiform hemangioendothelioma (KHE) and tufted angioma (TA). There is no consensus regarding intraoperative anticoagulation in patients with Kasabach-Merritt syndrome undergoing CABG. A man in his 70s with angina pectoris and lesions in the left main trunk (LMT) was scheduled for CAGB. He was diagnosed with Kasabach-Merritt syndrome as a child and had consumptive coagulopathy. There were concerns that using cardiopulmonary bypass and an antithrombin agent as an alternative anticoagulant might increase the risk of bleeding. Surgery was performed using an off-pump CABG (OPCAB) without heparin, and nafamostat was administered as an alternative anticoagulant at a bolus dose and continuously. The surgery was completed without thrombus formation or other adverse events, and the postoperative course was generally good. The graft was confirmed to be patent, and the patient was discharged on day 24. Nafamostat was thus found to be an effective intraoperative anticoagulant in a patient with Kasabach-Merritt syndrome. OPCAB with nafamostat represents a viable treatment option when heparin is contraindicated, but further studies are needed to clarify optimal intraoperative anticoagulation strategies.

## Introduction

In 1940, Kasabach and Merritt reported a case of a neonate with rapidly enlarging hemangioma and decreased platelet count, and this gave rise to the term “Kasabach-Merritt syndrome” [1], a condition associated with a hyperfibrinolytic system, thrombocytopenia, and consumptive coagulation abnormalities with kaposiform hemangioendothelioma (KHE) and tufted angioma (TA). Heparin is contraindicated in Kasabach-Merritt syndrome because it potentiates the effects of growth factors on cultured endothelium and mobilizes basic fibroblast growth factor from storage in extracellular matrix, and it can increase tumor size and worsen the disease [2]. In addition, heparin administration may exacerbate a tendency to bleed due to a consumptive coagulopathy. Coronary artery bypass grafting (CABG) is the first choice for managing coronary three-vessel lesions involving the left main trunk (LMT) according to the American College of Cardiology (ACC)/American Heart Association (AHA) guidelines [3]. During CABG, heparin is generally used to maintain anticoagulant status based on activated clotting time (ACT).

In cases where heparin is contraindicated, such as heparin-induced thrombocytopenia (HIT) [4], antithrombin agents such as bivalirudin and argatroban are often used as alternative anticoagulants. However, bivalirudin is not approved in Japan; meanwhile, argatroban, unlike heparin, has no specific antagonist, and its prolonged anticoagulant effect after cardiopulmonary bypass (CPB) withdrawal may lead to life-threatening bleeding. Therefore, in the present case, we opted for off-pump CABG (OPCAB) to avoid performing a surgery that required CPB, which necessitates a large amount of heparin or argatroban. In cases involving consumptive coagulopathy, there is a concern that argatroban may prolong bleeding tendencies, even during OPCAB. This necessitates the selection of an alternative anticoagulant.

Recently, several studies have reported the use of nafamostat in patients with heparin resistance during emergency cardiovascular surgery while using andexanet as a direct oral anticoagulant (DOAC) antagonist [5]; however, there have been no reports of CABG performed using nafamostat alone as the anticoagulant. In this report, we describe a case in which OPCAB was performed using only nafamostat as the anticoagulant in a patient with Kasabach-Merritt syndrome.

## Case presentation

**Figure 1:**
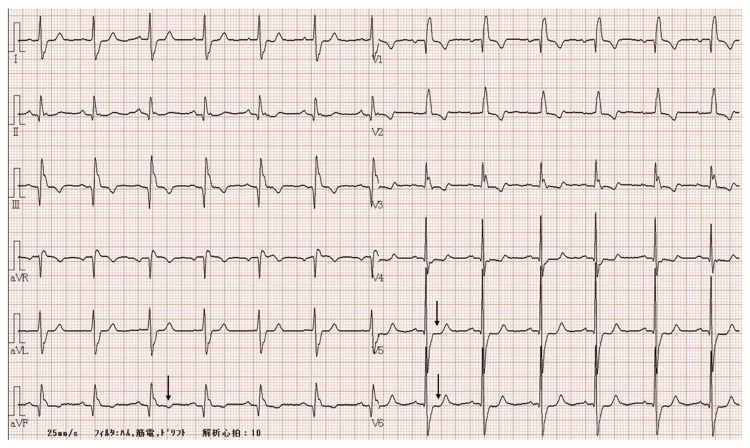
Preoperative electrocardiogram findings Sinus rhythm, complete right bundle branch block, and ST segment changes noted in leads aVf and V4-V6

The patient, a man in his 70s (height: 153 cm, weight: 57 kg), underwent preoperative echocardiography for a right inguinal hernia in January 2024, which revealed abnormal wall motion; thus, the patient was referred to the cardiology department. Coronary angiography revealed a three-vessel lesion involving the LMT, and the patient was referred for surgery and scheduled to undergo CABG. He had been diagnosed with Kasabach-Merritt syndrome owing to hemangiomas on the surface of the trunk since childhood. He underwent resection of hemangiomas in the left axilla and back. The patient was also on medications for hypertension, dyslipidemia, and hypothyroidism. On admission, his blood pressure was 127/65 mmHg, heart rate was 64 bpm, respiratory rate was 18 per minute, and body temperature was 36.5℃. Preoperative findings included a sinus rhythm of 64 bpm, aVf, V5, and V6 ST changes on electrocardiography (Figure 1), 39.6% ejection fraction on echocardiography, and no significant valvular disease. Coronary angiography showed 100% stenosis of the right coronary artery #2, 75% stenosis of the LMT, 99% stenosis of the left anterior descending artery, and 75% stenosis of the left circumflex artery #14. Blood test results revealed the following: hemoglobin, 12.9 g/dL; platelets, 102,000/mm^3^; activated partial thromboplastin time (APTT), 29 seconds; prothrombin time (PT), 13.9 seconds (international normalized ratio: 1.080); fibrinogen, 144 mg/dL; D-dimer, 14.05 μg/mL; fibrinogen degradation products, 34.5 μg/mL; and antithrombin III, 78% (Table 1).

**Table 1:** Laboratory blood tests before and after surgery PT-INR: prothrombin time-international normalized ratio, APTT: activated partial thromboplastin time, FDP: fibrin degradation products, ALT (GPT): alanine aminotransferase (glutamate-pyruvate transaminase), AST (GOT): aspartate aminotransferase (glutamic-oxaloacetic transaminase)

Test	Unit of measure	Before surgery	After surgery	Reference range
White blood cell	/µL	4,700	6,100	3,300-8,600
Hemoglobin	g/dL	12.9	9.9	13.7-16.8
Hematocrit	%	38.6	28.5	40.7-50.1
Platelet	×10^4^/μL	10.4	11.0	15.8-34.8
PT-INR	-	1.080	1.21	-
APTT	Seconds	29.1	35.1	24-39
Fibrinogen	mg/dL	144	139	200-400
FDP	µg/mL	34.5	-	0.0-5.0
D-dimer	µg/mL	14.05	-	0.00-1.00
Antithrombin Ⅲ	%	78	-	80-130
AST (GOT)	U/mL	24	21	13-39
ALT (GPT)	U/mL	16	12	10-42
Blood urea nitrogen	mg/dL	20	10	8-20
Creatinine	mg/dL	0.65	0.7	0.65-1.07
Creatinine kinase	U/mL	174	234	59-248
C-reactive protein	mg/dL	0.04	0.06	0.00-0.14

**Figure 2:**
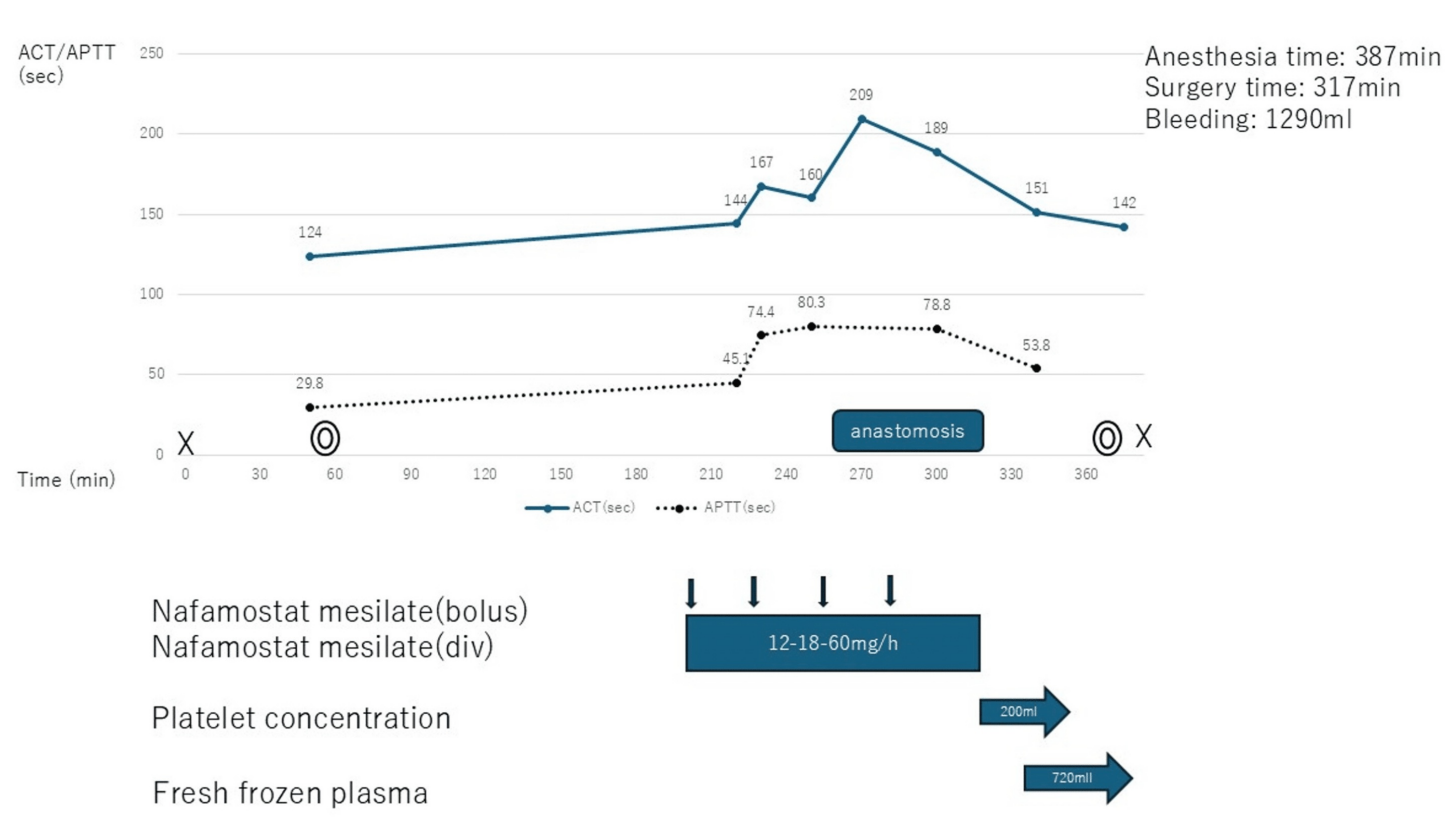
Chronological changes in ACT and APTT during the perioperative period The continuous infusion rate of nafamostat was initiated at 12 mg/h; however, ACT was not prolonged sufficiently. Nafamostat bolus administration was required until the ACT was sufficiently prolonged and to maintain ACT during anastomosis. During anastomosis, the continuous infusion rate of nafamostat was maintained at 60 mg/h. Continuous nafamostat administration was terminated, and ACT was promptly shortened. ACT: activated clotting time, APTT: activated partial thromboplastin time

Surgery was scheduled for OPCAB and left internal thoracic artery-left anterior descending anastomosis using nafamostat without heparin for anticoagulation. After inducing general anesthesia with midazolam, remifentanil, and rocuronium, anesthesia was maintained with sevoflurane, remifentanil, and fentanyl, and surgery was initiated. The ACT measured with kaolin did not reflect the effect of nafamostat because kaolin absorbs nafamostat [6]; therefore, the ACT was measured with Celite (Abbott Laboratories, Chicago, IL). The pre-intervention Celite ACT was 124 seconds. The left internal thoracic artery was embedded in and adhered to the sternum, which made harvesting the graft difficult. After harvesting the internal thoracic artery, with a target ACT value of 200 seconds, bolus (10 mg) and continuous (12 mg/h) administrations of nafamostat were initiated. Nafamostat administration was initiated at a dose of 0.2-0.3 mg/kg/h, which is considered relatively low. However, as the ACT and APTT were not prolonged sufficiently, the continuous dose was increased (12-18-60 mg/h). An additional bolus dose (120 mg total) was administered, after which the ACT (209 seconds) and APTT (80.3 seconds) were prolonged, and anastomosis to the coronary artery was initiated. After completing the coronary anastomosis and confirming anastomotic blood flow by Doppler ultrasound, continuous nafamostat administration was terminated, and red blood cell (RBC) concentrate, fresh frozen plasma (FFP), and platelet concentrate (PC) were administered. At the end of surgery, the ACT was 142 seconds. The patient was transferred to the intensive care unit (ICU) under sedation with propofol. The operation time was 5 hours and 17 minutes, and 560 mL of RBC, 720 mL of FFP, and 200 mL of PC were used. The total blood loss was 1,290 mL (Figure 2).

The patient's progress after admission to the ICU was generally good. During his stay in the ICU, the patient's platelet count ranged from 58,000 to 106,000/mm^3^, and the fibrinogen level increased from 139 to 291 mg/dL; the APTT ranged from 35.1 to 39.7 seconds, and the PT decreased from 15.4 to 13.7 seconds. No significant deterioration in coagulation ability was observed. The patient was transferred from the ICU to a regular room on postoperative day 7. The patient's progress was generally good, and graft patency was confirmed. He was discharged on day 24.

## Discussion

We reported a case of OPCAB using nafamostat alone as an anticoagulant in a patient with Kasabach-Merritt syndrome. Reports of cardiac surgery in patients with Kasabach-Merritt syndrome are rare, whereas reports on the resection of hepatic hemangiomas are more common [7]. Many patients with Kasabach-Merritt syndrome present with low preoperative platelet counts and coagulation abnormalities, necessitating measures against bleeding and coagulation disorders throughout the perioperative period [8]. Heparin administration is contraindicated in patients with Kasabach-Merritt syndrome because of the potential for increased incidence of hemangiomas in the setting of thrombocytopenia [2]. To the best of our knowledge, there are no reports on the safety of using heparin in the treatment of cardiovascular diseases in patients with Kasabach-Merritt syndrome.

CABG is the standard of care in patients with coronary artery disease, and heparin is commonly used as an anticoagulant during surgery. However, numerous studies have reported the use of argatroban for anticoagulation in patients with acute HIT type II and other conditions in which heparin is contraindicated. Several cases of OPCAB with effective anticoagulation using argatroban have also been described [9]; however, difficulties in controlling intraoperative and postoperative bleeding due to prolonged anticoagulation effects have been reported in patients undergoing CPB [10]. The patient had preoperative consumptive coagulopathy, and even with OPCAB, the excessive anticoagulant effect of argatroban may have made it difficult to control the bleeding.

Nafamostat is a serine protease inhibitor with anticoagulant properties, and it is used during hemodialysis and extracorporeal membrane oxygenation when heparin administration is difficult or contraindicated. Nafamostat has a very short half-time and multifactorial inhibitory effects on thrombin, complement, and fibrinosis [11]. There have also been reports of reduced heparin use in patients undergoing CPB [6,12], as well as maintenance of adequate anticoagulation with heparin in patients with heparin resistance following administration of andexanet as a DOAC antagonist [5].

In the present case, OPCAB with nafamostat was selected because heparin is contraindicated in patients with Kasabach-Merritt syndrome, and the patient's background of consumptive coagulopathy made it difficult to administer argatroban.

There is no consensus on the appropriate dosage of nafamostat for cardiac surgery until the ACT and APTT are prolonged. Based on the short ACT values from previous reports, the target ACT was set at 200 seconds [13]. Considering that CPB was not used and the concern of excessive anticoagulation, we initiated nafamostat administration at a low dose (approximately 12 mg/h). Reports on the appropriate dosage of nafamostat for achieving anticoagulation status vary [11]. In this case as well, we encountered difficulties in adjusting the bolus and continuous nafamostat doses until the patient was sufficiently anticoagulated. When nafamostat administration was interrupted after the anastomosis was completed, the ACT and APTT quickly returned to baseline values owing to the short half-life of nafamostat [11]. No clinical bleeding tendency during or after the procedure, graft thrombus, or other adverse events were observed; thus, OPCAB with nafamostat was effective in situations where heparin was not available. However, a study reported large amounts of thrombosis caused by using only nafamostat during cardiopulmonary procedures [14]. OPCAB reduces the risk of thrombosis compared with CPB settings because it allows the ACT value to be maintained at a lower level. Further studies are needed to determine the appropriate amount of nafamostat and the need for additional anticoagulants. Nafamostat provides less modulation of the anticoagulation status than heparin; it is thought to play a role as an anticoagulant in cardiac surgery, as with ECMO use [11].

## Conclusions

We encountered a case of OPCAB in which nafamostat alone was used as the intraoperative anticoagulant. Heparin is contraindicated in patients with Kasabach-Merritt syndrome; as our patient had Kasabach-Merritt syndrome and concomitant consumptive coagulopathy, we were reluctant to use argatroban during CABG. Therefore, OPCAB was performed using nafamostat. Although achieving adequate anticoagulation status during OPCAB with nafamostat monotherapy required additional time, no bleeding tendency or thromboembolic events were observed. Thus, in patients with coronary artery disease for whom heparin is contraindicated, OPCAB using nafamostat may represent a potential treatment option. However, since this report describes only a single case, the conclusions should remain cautious, and additional studies or case series are needed to confirm the safety and effectiveness of nafamostat in similar surgical settings.
